# Converting *Access Microbiology* to an open research platform: focus group and AI review tool research results

**DOI:** 10.1099/acmi.0.000232

**Published:** 2021-04-19

**Authors:** Alexandra M. Howat, Alexander Mulhern, Hilary F. Logan, Gaynor Redvers-Mutton, Chris Routledge, Justin Clark

**Affiliations:** ^1^​ Microbiology Society, London, UK

**Keywords:** open research platform, publishing, open science, preprints, society publisher, Wellcome Trust

## Abstract

The Microbiology Society will be launching an open research platform in October 2021. Developed using funding from the Wellcome Trust and the Howard Hughes Medical Institute (HHMI), the platform will combine our current sound-science journal, *Access Microbiology*, with artificial intelligence (AI) review tools and many of the elements of a preprint server. In an effort to improve the rigour, reproducibility and transparency of the academic record, the *Access Microbiology* platform will host both preprints of articles and their Version of Record (VOR) publications, as well as the reviewer reports, Editor's decision, authors' response to reviewers and the AI review reports. To ensure the platform meets the needs of our community, in February 2020 we conducted focus group meetings with various stakeholders. Using articles previously submitted to *Access Microbiology*, we undertook testing of a range of potential AI review tools and investigated the technical feasibility and utility of including these tools as part of the platform. In keeping with the open and transparent ethos of the platform, we present here a summary of the focus group feedback and AI review tool testing.

## Introduction

In July 2020, the Microbiology Society was awarded funding by the Wellcome Trust and the Howard Hughes Medical Institute as part of their Learned Society Curation Awards (https://wellcome.org/grant-funding/schemes/learned-society-curation-awards-closed). The awards support society publishers who ‘want to explore new ways of signalling the significance of published research outputs in an open and transparent manner’.

Our application proposed the conversion our existing sound-science journal, *Access Microbiology,* into an open research platform. The platform will offer the microbiology community a new publishing model that delivers greater peer-review transparency to help fast-track the communication of valuable research. The platform will feature artificial intelligence (AI) review tools to help authors improve their work, and the posting of article versions as preprints alongside their associated AI review reports, peer reviewer reports, Editor comments and author responses, to ensure a fully transparent peer-review process. Accepted articles will be typeset and published in their final, Version of Record form.

*Access Microbiology* welcomes articles from the entirety of microbiology and virology, and encourages the publication of replication studies, negative or null results, research proposals, data management plans, and additions to established methods. The publication criteria is based on methodological rigour rather than novelty. Converting the journal to an open research platform will increase transparency and openness, and therefore the rigour and reproducibility of the research.

During the first stage of this project the Society has been engaging extensively with our community regarding the platform, and performing initial technical assessments of the AI review tools and peer review software.

## Focus groups

### Recruitment and structure of the focus groups

To encourage debate and to ensure we listen to our community, we attempted to include a representative pool of various potential stakeholders, including published authors in *Access Microbiology*, Society members, authors published in other open research platforms, preprint users, early career researchers, open access specialists based at large universities, Wellcome-grant recipients, and Editors who currently serve on the *Access Microbiology* Board. An initial call for volunteers to be involved in the project was put out in July 2020 by direct email to Society members and in the Microbiology Society Newsletter. The list of respondents was used as the primary source of contacts to populate the focus groups. However, authors published in *Access Microbiology* were contacted based on their publications over the past 2 years, preprint users were selected from recent authors published in Society journals who submitted via a bioRxiv transfer process, Wellcome-funded authors were found via Wellcome’s publicly available list (https://wellcome.org/grant-funding/funded-people-and-projects), and open access specialists were already known to the Society from institutional agreements.

In advance of the focus group, participants were sent an overview of what the session would entail and were provided links and/or documentation to the AI review tools. In each session we presented the proposed model of the open research platform and asked for feedback. There were five to six participants in each group and three Microbiology Society staff members. Whilst each 90 min session mainly consisted of open discussion, the Chair guided the session through specific topics, including posing some open and closed questions. Topics included the overall model, open peer review, open data, and the AI review tools.

### Participants

Below are some key characteristics of each participant in each group. This information aims to provide context as to why they are an important stakeholder in the project. Limited information is provided to ensure anonymity is preserved.

#### Focus group 1

- Postdoctoral Researcher and member of the Early Career Microbiologists’ (ECM) Forum.
- Director of microbiology and Society member; Wellcome-funded.
- Senior Lecturer; Wellcome-funded.
- Senior Lecturer and virologist of over 40 years.
- Open access specialist at a large university.

#### Focus group 2

- Associate Professor and *Access Microbiology* author.
- Research Fellow, *Access Microbiology* author and user of bioRxiv.
- Group Leader, author published in other open research platforms, user of bioRxiv, Society member.
- Associate Professor, user of bioRxiv and Society member.
- Professor; Wellcome-funded.
- Open access specialist at a large university.

#### Focus group 3

- Principle Investigator and *Access Microbiology* author.
- Postdoctoral Researcher, member of the ECM Forum and *Access Microbiology* author.
- Postdoctoral Researcher and member of the ECM Forum.
- Group Leader, user of bioRxiv and Society member.
- Group Leader; Wellcome-funded.
- Lecturer; Wellcome-funded.

#### Editor focus group

Seven Editorial Board members of *Access Microbiology* were present: two Editor Mentors, three Editors and two Editor Mentees.

### The proposed model for the *Access Microbiology* open research platform

**Fig. 1.**
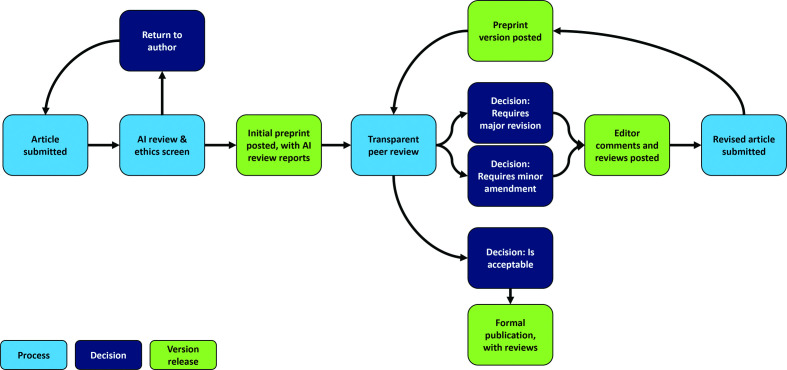
Proposed model of the *Access Microbiology* open research platform presented to the focus groups.

Participants were given a 10 min presentation contrasting a traditional journal publication model with the proposed model for the *Access Microbiology* open research platform (Fig. 1). In brief:

- Authors will submit their article to the platform, where it is assessed by the Editorial Office for policy and ethical compliance.
- In parallel, it will be run through a number of AI or machine learning review tools. The AI review tools under consideration and presented were SciScore™ (https://sciscore.com/mysubmission), Penelope (https://www.penelope.ai/), iThenticate® (https://www.ithenticate.com/) and Scholarcy™ (https://www.scholarcy.com/scholarcy-features/).
- The authors will then be given the opportunity to view the AI review reports and revise their article based on the results.
- Once the author resubmits their revised article, provided it complies with platform policy and ethical requirements, it will then be posted online as a preprint with a citable DOI, alongside the reports of the AI review tools.
- Meanwhile, the article will be assigned to an Editor and undergo transparent peer review, with two full reviews required.
- After receiving the reviews, the Editor will either recommend a ‘major revision’ or ‘minor amendment’ decision. Their decision and the accompanying reviewer reports will be posted on the platform.
- Once the authors revise and resubmit their article, the revised version and the authors' response to the reviewers will also be posted on the platform.
- If the Editor deems the article to be scientifically sound and of sufficient quality, the article will be accepted. The accepted article will then be fully typeset, copyedited and published as the final Version of Record.

### Perception of the overall model

Most participants across all groups (including Editors) liked the general idea of the model, and particularly praised its goal of increased transparency with respect to peer review, and as a platform that could help early career researchers both improve their work and get it published. One participant noted that this model has the potential to disrupt an established hierarchy of high-impact journals led by senior researchers, whilst another commented that it has the potential for wide dissemination.

There were concerns by a few participants across different focus groups that there is already a very well-established preprint community (e.g. bioRxiv, medRxiv) and that it might be difficult to convince authors to move away from these established platforms, especially when other successful sound-science journals such as *PLOS ONE* and *Scientific Reports* also exist. This was related to the perception of a lack of a unique selling point by some participants. The use of the terminology ‘posting’ and ‘publishing’ for preprints and Versions of Record, respectively, was well understood and felt to be important by some in highlighting when an article is truly published, as this is key for funding. A few participants in group 1 had concerns about reviewer fatigue if there was no option for Editors to reject, which was also extensively covered by the Editor focus group, described below.

After this initial and open discussion, when asked if they would consider publishing in the platform almost all participants across all focus groups indicated that they would. The exceptions included the two university open access specialists who are not microbiologists, one participant who was unsure and two who indicated that they would not because they do not typically publish in sound-science journals.

### Open versus transparent peer review

The concepts of ‘open review’ or ‘transparent review’ were introduced. In summary, reviewers’ names are published in open review, while transparent review involves publishing the content of the review but the reviewer can opt-out of having their name revealed. Almost all participants across all four groups agreed with the principle of open peer review and felt that it should be a goal to work towards. However, most participants had reservations about the real implications and felt strongly towards transparent peer review with an opt-in option. A few participants in group 2 were strongly in favour of open reviews, arguing that if a publisher launches an open research platform, everything should be truly open and that this would encourage better quality reviews. Two Editors highlighted that securing reviewers for *Access Microbiology* was already very difficult and were concerned that requiring reviewers to include their names would decrease the likelihood of people accepting to review, which was also noted as a possibility in focus group 2.

A larger discussion occurred in the Editor focus group regarding the poor quality of the reviews currently received in *Access Microbiology*, with multiple participants voicing concerns that posting these might risk the reputation of the platform and the Society itself. It was noted that reviewers often provide just one sentence, which is insufficient, and it was emphasized that there must be a way of dealing with these kinds of reviews on the platform. This led to a discussion on whether mandating open reviews would increase the quality and length of reviews, with two suggesting it might help; although one commented that the open reviews they had received on their own manuscript in an open review journal had not been much better and the Editor had allowed unfair comments to be sidestepped. Staff suggested the possibility of the platform launching with transparent (i.e., opt-in) reviews but that the goal could be to move towards a mandatory open review within 2 years, to allow for wider community adoption and understanding. One Editor responded that any change would have to be based on a survey of those who have both opted in and opted out, to see what the main reasons were for opting out.

### Open data policy

Many participants across the groups that discussed the open data policy (group 1 did not) also agreed with it in principle, with some arguing that it would be important for the platform to enhance transparency, and that it could support data reproducibility and null studies. However, it was suggested that this might disadvantage smaller and less well funded researchers because the data could rapidly be reused by others who are much better equipped, and could publish work on it before the article was accepted. It was highlighted that it is often cultural attitudes towards data sharing that prove to be a barrier, rather than operational considerations.

### Other themes covered in the editor focus group

#### Editors performing reviews

The Editor focus group was asked how they felt about Editors being asked to perform reviews on manuscripts handled by other Editors, as well as handling their own manuscripts, and how they felt about the Board being expanded to accommodate this new responsibility. Two Editors pushed back on this idea, stating that they were already overloaded with work, with one suggesting it would reduce the quality of the work they already do.

#### Editorial oversight and reputational concern

The Editors were posed the following question: if the model (in theory) allows authors to continually revise their article, at what point in the peer review process could or should it no longer be continued, and under what circumstances? All Editors felt strongly that there should be a route to a form of rejection for a variety of reasons. Three Editors felt that they should even be able to assess the work before it was posted and reject it at this point. The primary reason being that when articles are very badly structured and written, it can be impossible to know what has been done methodologically and therefore to scientifically assess it. Another major concern surrounded those articles that could be understood, but were completely scientifically unsound, and any form of revision would not resolve that fundamental issue. Of additional concern were scenarios in which two ‘Reject’ recommendations are received from reviewers on an article that Editors are not themselves an expert on, and how it would be difficult to see how they should not listen to these recommendations. There was a high level of concern from most Editors that allowing preprints on the platform with no prior screening by an Editor would cause the platform to be inundated with very poor preprints, risking the reputation of the platform and the Society.

When it was clarified that Editors would be able to make early ‘Major revision’ recommendations on articles that could not yet be sent out for review, Editors felt that this would increase the workload for themselves as they would essentially have to perform full reviews to make this decision. Three participants stressed that Editors should be trusted to make the final judgement. On the issue of authors being unable to return to their labs to perform more experimental work due to COVID-19 restrictions, one Editor said that this should not be an excuse; the work must always be sound. Another Editor felt that given the sound-science scope of *Access Microbiology*, Editors should not typically be asking authors to perform large amounts of experimental work, since the scope allows for incremental work, and if more work is being requested, it is a sign it probably is not sound and should be rejected. Another Editor disagreed, stating that it is possible to ask for experiments to make the work rigorous. One Editor also stated that they often ‘Rejected and invited resubmission’ for very poor language, but none had so far been resubmitted. Articles such as these would always remain on the new platform, again increasing the volume of poor-quality content.

Lastly, one Editor felt that the platform should not be a form of ‘repository’ and should not allow minimal research such as genome announcements, as Editors at a journal they had previously worked on had been inundated with articles containing research not sufficient enough to be published.

#### Editorial board structure

The Editors were asked whether a singular point of contact such as an Editor-in-Chief, as the other Society journals have, would be valuable to them for advising on ethical issues, appeals and edge-case articles. An Editor suggested that the previous idea of having one Editor Mentor and Mentee on the Society’s Publishing Panel to represent the platform could work. Both Mentors highlighted that the current Editor Mentor workload was relatively low and the current system of Mentors working with the other Board Members and the Journal Development Editor to resolve these situations worked well and that the journal was unique in this way. It was felt that, if there was to be a figurehead, they should be promoted from within the existing Board, but that it was not necessarily a requirement.

### AI review tools

There were some concerns from a few participants across the groups regarding the use of AI review tools. One concern related to asking authors to do more work during the submission process, and how this could be a barrier to submission. It was also raised that AI review tools can produce incorrect results, and having them used as a reason to reject an author could be unfair. Participants across all focus groups were concerned that publishing reports from any of the tools without clear guidance might be damaging if readers are not made fully aware of their possible weaknesses and the context required when interpreting them. Once participants were made aware that the tools would be primarily used by the authors to improve their work (even before preprinting), there was greater approval of their use, if handled appropriately by the Society.

#### SciScore™

Participants were asked to discuss the sample SciScore™ report, as shown on the SciScore™ website, https://www.sciscore.com/media/Sample-Report.pdf. Many participants across all groups saw the potential for this tool, with the greatest emphasis placed on allowing authors to improve the rigour of their work and spot mistakes they would not have previously known required correction. Others stated it would help reviewers to spot ethical and data availability issues, whilst one suggested it could help highlight the use of different sequencing technologies. Whilst a couple of participants stated they would not have time to read the report alongside the preprint, one felt strongly that providing accurate and detailed methodology was useful when trying to reproduce a paper’s work. There was no indication that it would be useful to Editors. As noted above, it was stressed by many participants that clear guidance would be needed if the report were to be published with the article.

#### iThenticate®

Participants were asked to discuss a sample file sent to them by the Society. Concerns about iThenticate® were centred once again around publishing reports without providing clear guidance, with the open access specialist in group 2 explaining that misinterpretation of the report was a frequent issue with their students, which led to a lot of distress. Once participants were made aware that authors and Editors would be able to access the full online version before the posting of preprints, many saw the value in this tool in allowing an author to improve their work. Examples included: researchers not realizing they are self-plagiarizing due to years of writing in the same style; poor scholarship (incorrect referencing); and authors being able to spot when sentences had been lifted from previously published work because co-authors had written large parts of the manuscript. A point raised in multiple groups was that it is notoriously difficult to rewrite some methods that are well established (e.g. DNA extraction) and that allowances for slightly higher ‘scores’ in areas like this should be given, with one participant commenting that there would be an irony in a platform aiming to be transparent, but penalizing authors for attempting to ensure their methods were detailed and accurate.

#### Penelope

Participants were asked to discuss the example article on the Penelope.ai website, https://app.penelope.ai/submissions/demo/?role=author. Penelope was considered a useful tool for authors by some in each group, with a couple independently commenting that it would be particularly useful for early career researchers in guiding them how to properly construct a manuscript and self-improve. A few others stated that it was useful in simply checking they had complied with journal policies, with one saying it looked like the useful linting software they regularly use to check their code, whilst another liked that it would pick up on missing figure citations or references. A concern was raised by a couple of participants that this tool might be used to enforce archaic formatting requirements.

#### Scholarcy™

Participants were asked to discuss the flashcard generator on the Scholarcy™ website, https://app.scholarcy.com/flashcard-generator.html. This tool was regarded as the least useful of the tools by many participants across the groups, with many commenting they would not use it. Two participants explained that they had run one of their manuscripts through it and it had produced completely irrelevant results. A couple of participants also commented that as a reviewer they would not be confident that it was completely accurate and they would end up using the raw manuscript anyway. It was suggested that it might be useful for authors in writing a press release if it was free for the Society, but otherwise it was not useful.

### Caveats and limitations of the focus groups

Whilst we attempted to include a representative and diverse set of stakeholders, we recognize that our selection and invitation process using primarily a list of contacts who expressed interest through Society membership and activities likely captures only a subset of potential stakeholders. For example, most participants were based in the UK, whilst only approximately 5 % of the corresponding authors of articles submitted to *Access Microbiology* are based in the UK. We tried to ensure that authors who have published in *Access Microbiology* were well represented in the groups, but due to three of these invited participants being unexpectedly unable to attend, there were only four in total who took part. We also appreciate that the very nature of inviting participants to discuss an open research platform likely skews the eventual participant pool in favour of people already knowledgeable or interested in the concept and may therefore be more likely to have viewpoints on the model.

## Testing and technical feasibility of AI review tools

To ensure that the AI review tools are appropriate for the content published on the *Access Microbiology* platform, articles were run through each tool and the results analysed, including accepted and rejected articles, and various article types. Tools were also investigated with respect to their possible integrations with our peer review system, Editorial Manager®, and how much manual intervention would be required by Editorial staff on a per article basis. Below is a summary of these tests.

### SciScore™

#### Testing

SciScore kindly gave us an account to test the software. Methods, ethics statements, data availability statements and any other relevant information in each article was run through the tool and the score recorded, as well as how accurately and fairly it detected criteria. On average, accepted articles had a higher score (2.78, *n*=18) than rejected articles (2.33, *n*=6)., with the known average SciScore™ of an article in PubMed Central being 4.2 [1]. By nature, Case Report article types do not have a methods section as they describe the retrospective treatment of a patient, so the full case description was included for testing. For these article types, the tool would often detect that the article contained work with an individual but then incorrectly expected other related criteria, such as blinding, randomization, attrition etc. After discussion with SciScore, they confirmed that Case Reports were not an article type that had been included in the development of the tool.

For many Research Articles, sequencing and software were often correctly detected and clearly did not include sufficient information such as accession numbers, whilst many articles often omitted replication, power analysis and many research resource identifiers (RRIDs) that could easily be included by the authors. ‘Not detected’ was consistently present for all human-related checks in the Rigour Adherence Table on articles where this was completely irrelevant. After discussion with SciScore, this was determined to be a bug in the tool and would be fixed very soon. However, based on our recommendations, they stated that they are now considering rewording ‘Not detected’ to ‘Not relevant’ in instances when the tool does not expect a criterion, to highlight to authors when they do not need to address these areas.

#### Technical feasibility

SciScore are in the process of a full integration with Editorial Manager®, with the expectation that it will be included in the next version release. Authors will manually paste their own methods during the submission process and the report will be run automatically when they submit, which returns the full set of results to the system. Authors, staff, Editors and reviewers should all be able to access the report, if given permission to do so. With a small amount of manual work by Society staff, the report can easily be included in the Manuscript Exchange Common Approach (MECA; https://manuscriptexchange.org/) export to be posted online alongside the preprint.

### iThenticate®

#### Testing

Testing was done using the Microbiology Society’s established account with Turnitin. Overall similarity scores were noted, and individual matches were checked to assess relevance. On average, accepted articles scored 15.3 (*n*=10) whilst rejected articles scored 32 (*n*=9), suggesting that either peer review help improved authors’ work or that authors who are accepted are more careful with their writing. Most individual matches on accepted articles were only 1–2% and linked to very short and mandatory pieces of text, such as affiliations, standard funding statements, or commonly accepted phrases within the field. However, even some of the accepted articles contained longer sentences that were identical to previous work and should be rewritten. Some articles that had already been published were also checked, revealing that whilst the tool did pick up the published article, it often only matched with the abstract or only some parts of the rest of the manuscript.

#### Technical feasibility

iThenticate® is already fully integrated with Editorial Manager®. Staff, authors, Editors and reviewers can be given full access to the interactive online results, as well as the PDF report, if required. As with SciScore™, the report can easily be included in the MECA export to be posted online alongside the preprint.

### Penelope

#### Testing

Penelope was tested using the freely available precheck tool on their website: https://www.penelope.ai/precheck. Penelope has no score-based output, so the tool was scored as ‘Useful’ or ‘Not useful’ for each article based on the correct detection of key aspects of articles. The tool was considered ‘Useful’ 63 % of the time (*n*=19), and it is worth noting that these articles had already been screened by in-house staff, so it is assumed that the tool would have picked up more errors than we found if run on unchecked articles. It was useful in detecting missing references (or citations for references), missing figure legends (or figure citations) and ethical statements. Notably, it was able to recommend that authors consult the CARE checklist (https://www.care-statement.org/checklist) for Case Reports. However, because it has been designed to specifically expect authors to provide funding and conflict of interest statements under these respective headings, it did not detect any that did not fall under these, even if the statements themselves were present in the manuscript. The file size limit is 10 MB, which prevented a couple of articles from being assessed.

#### Technical feasibility

Penelope is not integrated in Editorial Manager® and at the time of writing, there were no confirmed plans for this to happen soon, although an API is available. However, the tool can easily be used by authors as a standalone product (e.g., from within our website) before submitting to Editorial Manager®, although the Editorial Office, Editors and reviewers would not receive any of these results associated with a submitted manuscript.

### Scholarcy™

#### Testing

Scholarcy™ was tested using the freely available flashcard generator on their website, https://app.scholarcy.com/flashcard-generator.html?web=1&wdLOR=cA57D6039-E93C-4287-92DC-5BD2945568D5. The tool also has no score-based output so testing was based on its ability to generate useful lay summaries and highlights, and detect relevant keywords. Only seven articles were tested (all accepted) but only one was considered ‘Useful’. The main issues included the Tweet/lay summary frequently missing key information that rendered it meaningless, and full stops used in abbreviated bacterial names (e.g. *B. subtilis*) often being detected as the end of a sentence, producing stunted and useless summaries.

#### Technical feasibility

Scholarcy™ is not integrated in Editorial Manager® and at the time of writing, there were no confirmed plans for this to happen soon, although an API is available. Like Penelope, the tool can be used by authors as a standalone product (linked within our website) before submitting to Editorial Manager®, but the Editorial Office, Editors and reviewers would not receive any of these results associated with the submitted manuscript.

## Outcome and next steps

These findings and some recommendations were presented to the Project Steering Group in March 2020. However, one of our main recommendations was to try and capture more feedback from our community on the more divisive aspects of the model, such as open peer review and the open data policy. We will do this by running a short survey over the coming months.
